# Cognitive and emotional conflict control among children affected by parental HIV/AIDS in China

**DOI:** 10.3389/fpsyt.2026.1742261

**Published:** 2026-03-04

**Authors:** Huihui Wang, Juan Liu, Danfeng Wen, Wan Zhao, Xiaoming Li, Junfeng Zhao

**Affiliations:** 1 School of Humanities, Henan Kaifeng College of Science Technology and Communication, Kaifeng, China; 2 Mental Health Education Center, Yellow River Conservancy Technical University, Kaifeng, China; 3 Institute of Behavior and Psychology, School of Psychology, Henan University, Kaifeng, China; 4 School of Psychology, Nanjing Normal University, Nanjing, China; 5 Department of Health Promotion, Education, and Behavior, University of South Carolina, Columbia, SC, United States

**Keywords:** children affected by parental HIV/AIDS, China, cognitive conflict control, emotional conflict control, stroop task

## Abstract

**Background:**

Children affected by parental HIV/AIDS constitute a particularly vulnerable population, often facing heightened risks in psychosocial adaptation due to family disruption, social stigma, and resource deprivation. However, the characteristics of their executive functions, particularly the core component of conflict monitoring, remain unclear.

**Methods:**

The present study compared the performance of children affected by parental HIV/AIDS (n=32) to a control group (n=32) on a cognitive conflict and emotional conflict face-word Stroop task.

**Results:**

The results of the cognitive Stroop task demonstrated that both groups showed better performance in congruent stimuli compared to incongruent stimuli (86.20% vs.67.30%; 568.43ms vs.586.04ms). In the emotional Stroop task, the control children displayed a significant interference effect both in the accuracy (85.70% vs. 70.70%) and reaction time (581.53ms vs. 597.98ms) to the incongruent condition compared to the congruent condition. However, the Stroop effect was absent in the children affected by parental HIV/AIDS. The children affected by parental HIV/AIDS can monitor cognitive conflicts, but show some deficits in monitoring emotional conflicts.

**Conclusion:**

In addition, the present findings suggest that the deficit of the emotional conflict control ability among the children affected by parental HIV/AIDS might be due to the fact that emotional stimuli occupy more cognitive resources in the emotional conflict monitoring process.

## Introduction

Children affected by parental HIV/AIDS refer to children younger than 18 years old whose parents are living with HIV/AIDS or die of HIV/AIDS (1). This population is recognized as particularly vulnerable (2). Their vulnerability arises not only from disease-related stigma but also is deeply rooted in a systemic adversity characterized by lack of care, deprivation of economic and educational resources, and chronic multiple psychological stressors (3, 4). Extensive research indicates that such early-life adversity can significantly impair the development of executive functions (EFs) in children (4–6). Given that EFs are core to self-regulation and social adaptation (7, 8), deficits in these areas may further entrench developmental disadvantages, potentially creating a vicious cycle that exacerbates difficulties in academic achievement, emotional regulation, and future planning.

Conflict control is a crucial component of cognitive control within EF. Conflict control monitors conflict in perceptual inputs, detects conflict between one’s preferred and required responses, and executes conflict resolve over these conflicts (9, 10). Conflict control is demonstrated by rapid detecting conflict before and after behavioral responses and adjusting cognitive resources to alter subsequent performance when performing tasks in which there is competition between task-relevant and task-irrelevant information (11, 12). An example of this type of task is the Stroop task (13), where participant reaction time (RT) is longer when incongruent information is presented than when congruent information is presented. This additional time, known as the Stroop effect, is considered to reflect the detection of conflicting information, many times through the use of colors and words (14, 15).

Importantly, Conflicts not only exist in the cognitive domain, they also reside in the emotional domain (16). In the real world, our thoughts, reactions and decisions are impacted by the presence of emotional stimuli. Therefore, it is important to know how cognitive and emotional processing interact in the detection of conflicts (17). Past research has investigated both emotional and non-emotional control mechanisms using the face-work Stroop task in order to equate cognitive and emotional conflict (18). In one study, researchers used event-related potentials (ERP) and other advanced technology to study the influence of emotion on cognitive control, and found an interaction between emotion and cognitive control (19). Positive emotion is associated with the broadening of attentional scope, relaxation and well-being, which further promotes the development of cognitive control. Most studies examine the impact of negative emotion on cognitive control have reported impaired task performance (20, 21), whereas others have showed enhanced effects of negative emotion compared to neutral stimuli, or even null effects (22, 23). This suggests that the negative emotion may differentially influence task performance when emotion interacts with cognitive control.

More deficits in conflict tasks demonstrated by longer reaction times and increased errors have been reported among vulnerable groups compared to control participants (24–26). For example, Xue examined the conflict control performance of treatment-resistant depression patients (TRD) with an emotional Stroop task, finding that TRD patients showed significantly slower RT compared to healthy controls. In addition, another study compared the performance of children with attention deficit hyperactivity disorder (ADHD) and healthy children in cognitive conflict tasks, demonstrating that the accuracy rate of ADHD children is generally lower than that of the healthy children, while there was no significant difference on the reaction time between them (25). Despite these insights, few studies have specifically investigated conflict processing—and the distinct contributions of cognitive versus emotional conflict—in children affected by parental HIV/AIDS.

Indeed, existing research on children affected by parental HIV/AIDS has predominantly focused on documenting their psychosocial risks, such as elevated levels of depression, anxiety, and externalizing behaviors (27), or on assessing their broader cognitive profiles and academic challenges (28). However, there remains a lack of fine-grained, experimental examination of their core cognitive control processes, particularly those underlying conflict monitoring and resolution. By isolating cognitive and emotional conflict, the present study can provide a more precise understanding of the specific patterns and mechanisms underlying EF impairments in these children, thereby contributing to theories on how early adversity shapes cognitive development.

Therefore, the aim of the present study was to explore the characteristics of cognitive and emotional conflict in children affected by parental HIV/AIDS, and to investigate the influence of emotion on the process of conflict control. Based on the findings of previous research, we predicted that the children affected by parental HIV/AIDS would demonstrate the cognitive conflict, but show some difficulties in the emotional conflict monitoring process.

## Methods

### Participants

We performed a sample size calculation on the basis of G∗Power3.1, using an alpha level of 0.05 with 95% power to detecta large effect size (f = 0.4). Results showed that a sample size of 12 would be needed to assure the adequate statistical power. The participants were recruited from rural communities in economically underdeveloped areas of central China. They had no life−threatening illnesses and were free of major chronic diseases including HIV/AIDS. The experimental group included 32 children affected by parental HIV/AIDS (17 boys, age:12.86 ± 0.66 years) and the control group included 32 children not affected by parental HIV/AIDS (16 boys, age:13.05 ± 1.04 years). All the subjects had a normal or adequately corrected vision, were right-handed, and reported no history of mental, medical, or neurological disorders. At the end of the experiment, they received an age-appropriate gift as a token of appreciation. Written informed consent was obtained for the study.

### Experimental procedure

All participants performed two sets of formal experiments, relating to the recognition of gender and emotion in faces selected from the Chinese Facial Affective Picture System (CFAPS) (28). The arousal values evoked by the positive and negatives in Experiments 1 and 2 were not significantly different. Task presentation and behavioral response recording were performed using E-prime 2.0 software. To familiarize themselves with the experiments, each participant was asked to practice trials before completing the formal experiments. Each participant completed 20 practice trials before the formal experiments to familiarize themselves with the task. All participants completed both the cognitive and emotional Stroop tasks.

### Experiment 1

In Experiment 1, a cognitive face-word Stroop task was used to elicit cognitive conflict. The target stimuli included 40 pictures of neutral facial expressions (20 females, 20 males) similar in size, brightness, contrast grade, and other physical properties obtained from the CFAPS. In the task, either a Chinese character for male (“男”) or female (“女”) was superimposed across each face in red font. The words and facial expressions were either congruent (e.g., character meaning male superimposed onto a male face picture), or incongruent (e.g., character meaning male superimposed onto a female face picture).

**Figure 1:**
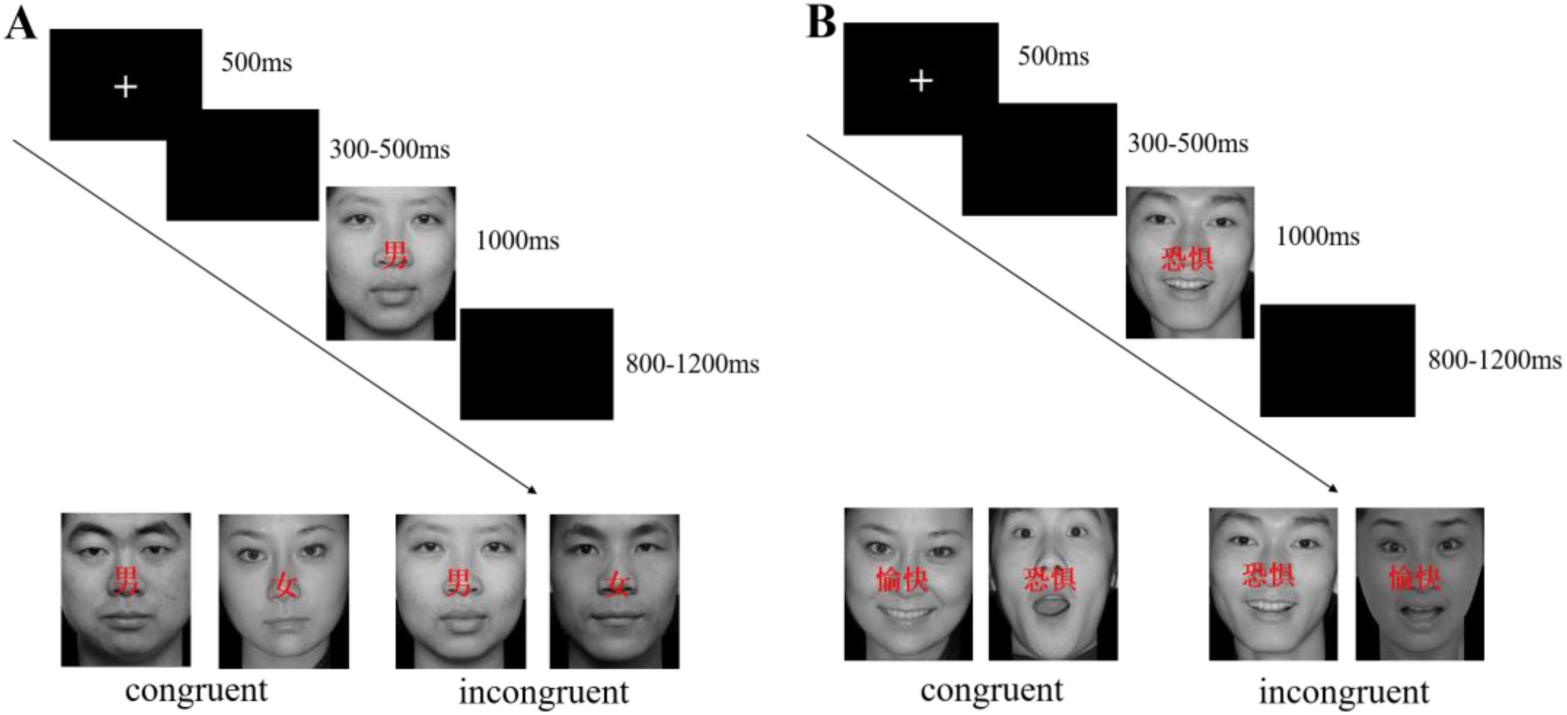
Procedure of Experiments 1 **(A)** and 2 **(B)**.

Each trial began with a fixation “+” for 500 milliseconds (ms), followed by a blank screen lasting randomly from 300 to 500 ms. After the blank screen, children saw a neutral face with a gender word on the screen (**Figure 1A**) and they were asked to identify the gender of the target and to select the response probe as quickly and correctly as possible by pressing either a “A” or “L” button using the left or right thumb on a computer keyboard. If they did not respond within 1000ms, the picture would disappear and their response would be coded as missing. If children gave a correct response, the next trial would occur between 800-1200 ms (Mean 1000ms). There was a total of 240 trials, with 60 for each condition.

### Experiment 2

In Experiment 2, we used an emotional face-word Stroop task to elicit emotional conflict. The target stimuli included 20 pictures of fear expressions (10 females, 10 males) and 20 pictures of happy expressions (10 females, 10 males) obtained from the CFAPS. Emotional faces (either happy or fear) were placed in the center of a screen and either a congruent (e.g., happy face with the character “快乐”, which means “happy”) or incongruent (e.g., happy face with character “恐惧”, which means “fear”) Chinese character was superimposed on the face at approximately the location of the nose.

Participants were asked to judge the valence of the emotional faces as quickly and correctly as possible by pressing either a “A” for fear or “L” for happy using the left or right thumb on a computer keyboard. In this experiment, the stimulus was presented in the same way as in Experiment 1 (**Figure 1B**). Experiment 2 also contained four blocks and each block had 60 trials for a total of 240 trials. The trial types assigned to different task conditions were pseudo-randomly assigned.

### Statistical analysis

Data were analyzed using one-way repeated-measures analysis of variance (ANOVA), with accuracy and reaction time (in milliseconds) as dependent variables, group (the children affected by parental HIV/AIDS vs control group) as between-subjects factor, and condition (i.e., congruent vs. incongruent) and task (i.e., cognitive Stroop vs. emotional Stroop) as within-subjects factor. All analyses were conducted using SPSS22.0.

## Results

### Cognitive conflict characteristics among children affected by parental HIV/AIDS

*Accuracy*. As shown in **Table 1**, there was a significant main effect of condition (F = 32.82, *P* < 0.001, *η*² = 0.35) on accuracy (i.e., proportion of correct responses), with participants demonstrating significantly less accuracy in the incongruent condition (67.31%) than in the congruent condition (86.23%). The main effect of group was also significant (F = 12.07, *P* < 0.01, *η*² = 0.16) with the experimental group (71.21%) demonstrating less accuracy than the control group (82.29%).

**Table 1:** Mean accuracy and reaction time of Experimental and Control Groups in cognitive Stroop task (*M* ± *SD*).

	Congruent	Incongruent	F_GROUP_	F_CON_	F_GROUP*CON_
ACC (%)					
Total	86.23 ± 13.75	67.31 ± 23.51	12.07^**^	32.82***	2.35
Exp Group	83.13 ± 17.43	59.31 ± 26.46			
Con Group	89.21 ± 7.62	75.45 ± 16.93			
RT (ms)					
Total	568.43 ± 72.21	586.04 ± 77.85	0.16	22.80***	5.76*
Exp Group	569.14 ± 85.62	577.90 ± 87.92			
Con Group	567.71 ± 57.14	594.18 ± 66.70			

*p < 0.05, **p < 0.01, ***p < 0.001.

*Reaction Time*. There was a significant main effect of condition with the reaction time (in milliseconds) with participants demonstrating significantly faster RTs which means improved performance during the congruent condition (568.43ms) than the incongruent condition (586.04ms; F = 22.80, *P* < 0.001, *η*² = 0.27). The interaction between group and condition was significant (F = 5.76, *P* < 0.05, *η*² = 0.08), with the control group demonstrating significantly faster RTs during the congruent condition (569.14ms) than the incongruent condition (594.18ms) while the experimental group did not demonstrate significantly faster RTs to the congruent condition when compared to the incongruent condition.

### Emotional conflict characteristics among children affected by parental HIV/AIDS

#### Accuracy

A 2 (Group) × 2 (Condition: congruent vs. incongruent) mixed ANOVA was conducted for group comparisons (See **Table 2**). There was significant main effect of condition (F = 36.65, *P* < 0.001, *η*² = 0.37), with the congruent condition (79.62%) being significantly more accurate than the incongruent condition (59.78%). The main effect of group was also significant (F = 23.81, *P* < 0.001, *η*² = 0.28) with the experimental group demonstrating less accuracy than the control group (61.11% vs. 78.24%). There was no significant interaction between group and condition on accuracy for the emotional Stroop task.

**Table 2:** Mean accuracy and reaction time of Experimental and Control Groups in emotional Stroop task (*M* ± *SD*).

	Congruent	Incongruent	F_GROUP_	F_CON_	F_GROUP*CON_
ACC (%)					
Total	79.58 ± 16.41	59.70 ± 24.81	23.81***	36.65***	2.18
Exp Group	73.42 ± 20.43	48.82 ± 26.94			
Con Group	85.72 ± 7.54	70.71 ± 16.53			
RT (ms)					
Total	567.50 ± 76.10	570.77 ± 90.43	4.37*	0.44	7.16*
Exp Group	553.47 ± 92.45	543.57 ± 107.46			
Con Group	581.53 ± 53.06	597.98 ± 59.51			

*p < 0.05, ***p < 0.001.

#### Reaction time

As shown in **Table 2**, the main effect of group was statistically significant (F = 4.37, *P* < 0.05, *η*² = 0.07), with the experimental group (548.52ms) demonstrating significantly faster RT than the control group (589.75ms). The interaction between group and condition was significant (F = 7.16, *P* < 0.05, *η*² = 0.10). The control group demonstrated significantly faster reaction times during the congruent condition (581.53ms) when compared to the incongruent condition (597.98ms), while there was no significant difference between the two conditions among the experimental group.

### Effects of emotion on conflict control of children affected by parental HIV/AIDS

#### Accuracy

A 2 (Group) × 2 (Task: cognitive Stroop vs. emotional Stroop) mixed ANOVA was conducted for group comparisons (See **Table 3**). The main effect of task was significant (F = 20.82, *P* < 0.001, *η*² = 0.25) with the cognitive Stroop task (76.82%) being significantly more accurate than the emotional Stroop task (69.73%). There was a significant main effect of group (F = 22.57, *P* < 0.001, *η*² = 0.27), with the experimental group demonstrating less accuracy than the control group (66.14% vs. 80.19%). The interaction between group and task was not statistically significant (F = 3.65, *P* = 0.06, *η*² = 0.06). A pairwise comparison of task indicated that compared to the cognitive Stroop task (71.23%), the emotional Stroop task (61.14%) showed significantly less accuracy among the children affected by parental HIV/AIDS (F = 20.96, *P* < 0.001, *η*² = 0.25), while there was no significant difference between the two tasks among the control group (82.31% vs. 78.24%, F = 3.52, *P* = 0.06, *η*² = 0.05).

**Table 3:** Effects of emotion on conflict control of children affected by parental HIV/AIDS.

	Cognitive	Emotional	F_GROUP_	F_TASK_	F_GROUP*TASK_
ACC (%)					
Total	76.84 ± 13.90	69.61 ± 16.33	22.57***	20.82***	3.65
Exp Group	71.22 ± 15.23	61.14 ± 17.15			
Con Group	82.35 ± 9.83	78.26 ± 10.07			
RT (ms)					
Total	577.23 ± 73.51	569.14 ± 80.98	2.04	0.87	3.09
Exp Group	573.52 ± 84.97	548.52 ± 97.31			
Con Group	580.95 ± 61.09	589.75 ± 54.60			

***p < 0.001.

#### Reaction time

As shown in **Table 3**, there was not statistically significant interaction between group and task (F = 3.09, *P* = 0.06, *η*² = 0.05). The experimental group demonstrated significantly faster reaction times (548.52ms) in the emotional Stroop task compared to the cognitive Stroop task (573.52ms), but the two tasks did not significantly differ in RTs among the control group (580.95 ms vs. 589.75 ms), (F = 0.52, *P* = 0.48, *η*² = 0.01).

## Discussion

The primary aim of this study was to examine cognitive control functioning - specifically conflict monitoring - in the children affected by parental HIV/AIDS. We used the gender word-face Stroop task and emotional word-face Stroop task to measure cognitive conflict and emotional conflict. The results suggest that they exhibit specific difficulties in processing emotional conflicts and in their overall cognitive control strategies. This pattern reflects the complex impact that early systemic adversity may have on the development of their executive functions.

Consistent with prior evidence (29), children affected by HIV/AIDS exhibited lower accuracy across cognitive Stroop tasks and emotional Stroop tasks compared to controls. This generalized impairment may be attributed to the chronic and multifaceted adversity characterizing their life circumstances, including poverty, parental illness or loss, and social stigma, all of which are known to detrimentally affect mental health (30). In addition, Prolonged exposure to such stressors can dysregulate the hypothalamic-pituitary-adrenal (HPA) axis, potentially impairing the prefrontal cortex—a neural substrate critical for executive function and conflict monitoring (31). This neurobiological pathway offers a plausible explanation for the weakened cognitive control “hardware” observed. Notably, similar performance deficits—characterized by significantly reduced accuracy in the experimental groups compared to controls—have also been documented in other vulnerable populations exposed to early adversity, such as deaf children and left-behind children (32, 33). This suggests that chronic stress may impair developing cognitive control systems through a common underlying pathway (34, 35).

An intriguing secondary finding was the demonstration of a distinct response pattern: affected children responded faster but less accurately than controls, particularly in the emotional Stroop condition. This pattern reflects a strategic speed-accuracy trade-off (36). Chronic exposure to unpredictable and stressful environments may foster a cognitive style that prioritizes rapid responding over deliberate, accurate processing—a tendency possibly linked to stress-related alterations in prefrontal circuits governing impulse control. Within the emotional task, the heightened resource demands of processing affectively charged faces may further deplete the cognitive capacity available for response inhibition and accuracy checks. This propensity to trade accuracy for speed could stem from transient anxiety induced by the testing context or may constitute a more enduring, adaptive coping strategy honed in persistently high-pressure environments (37, 38). A similar behavioral profile has been noted in deaf children, reinforcing the notion that early adversity can shape characteristic response strategies (32).

A comparison between the cognitive and emotional Stroop tasks revealed that children affected by parental HIV/AIDS displayed lower accuracy in the emotional Stroop condition relative to the cognitive one. This pattern provides support for the limited cognitive resources theory (39). For these children, the emotional task likely triggers high-load “emotion-cognition conflicts” that are closely tied to their lived experiences, thereby consuming greater psychological resources (40). This results in insufficient resources for subsequent conflict detection and resolution, exacerbating interference effects under emotional conditions. Additionally, research has indicated that children affected by HIV/AIDS demonstrate an attentional bias toward emotional faces. During the processing of emotional facial stimuli, their attention is involuntarily and persistently captured by such cues. These observations collectively point to a core deficit in their capacity to regulate emotionally salient distractions and flexibly allocate cognitive resources when confronted with emotionally challenging situations (41). This bias towards emotion stimuli finds parallels in research on children with hearing impairments, who also show heightened attention to emotion information (42).

In summary, this study preliminarily indicates that children affected by parental HIV/AIDS show clear deficits in emotional conflict processing, along with distinct cognitive resource allocation and response strategies. These characteristics suggest that early systematic adversity may selectively impair higher-order control functions involving emotion-cognition interactions, rather than basic conflict detection abilities. Future research should employ neuroscientific techniques to validate the underlying neural mechanisms and explore whether targeted cognitive training or psychological interventions can enhance emotional conflict processing efficiency and cognitive control strategies, thereby providing empirical evidence to disrupt the cycle of developmental disadvantage.

## Data Availability

The raw data supporting the conclusions of this article will be made available by the authors, without undue reservation.
